# Impact of Rotation Timing on Emergency Medicine Sub‐Internship Course Outcomes

**DOI:** 10.1002/aet2.70227

**Published:** 2026-07-23

**Authors:** Sofia Tuttle, Allison Beaulieu, Rowan Kelner, Christine Raps, Robert Stephen, Julia Ruggieri, Emad Awad, Brian Merritt, Patrick G. Hughes

**Affiliations:** ^1^ Department of Emergency Medicine, Spencer Fox Eccles School of Medicine University of Utah Salt Lake City Utah USA

## Abstract

**Background:**

Emergency medicine sub‐internships traditionally occur in summer months to facilitate letters of recommendation for residency applications, although it is unknown whether sub‐internship timing impacts course outcomes. The purpose of this study was to identify seasonal trends in course outcomes.

**Methods:**

This retrospective observational study examined 84 EM‐bound medical students during a 4‐week EM sub‐internship between 2021 and 2024. During the rotation, students received on‐shift evaluations graded on a Likert scale from 0 (inadequate) to 4 (outstanding) and completed an online SAEM exam (scored 0–100), both of which contributed to the final course grade. Course outcomes included the number of evaluations, average evaluation score, SAEM score and final course grade. Outcomes were compared between students rotating in May–July (25 students) and August–October (59 students). Analysis was completed in SPSS using independent *t*‐tests, Mann–Whitney *U* tests and regression analysis to adjust for confounders.

**Results:**

There were no significant differences in outcomes between the May–July and August–October subgroups when comparing average evaluation score (*p* = 0.11), SAEM score (*p* = 0.32) and final course score (*p* = 0.21).

**Conclusion:**

No performance differences were observed between early summer (May–July) and fall (August–October) rotations. These findings suggest that early summer rotations do not offer a significant advantage over fall rotations. Limitations include a single site study. Future research is needed to investigate factors contributing to seasonal variation in sub‐internship performance.

## 1 Introduction

Emergency medicine (EM) sub‐internships are an essential step in the path to applying for an EM residency and are traditionally concentrated in the summer months. As residency applications are due in September, many students preferentially schedule their sub‐internships during the summer and early fall months to ensure that letters of recommendation can be submitted before applications are released to programs. In contrast, some students consider the impact of more clinical experience, and may preferentially schedule their sub‐internship later in the year to optimize their EM knowledge prior to this ‘audition’ rotation [1]. While there are typically more away rotation spots available than students seeking them, peak‐season sub‐internship spots fill quickly, leaving many off‐peak rotation spots available [2]. As students typically need four to five applications to receive one sub‐internship offer, off‐peak rotation times offer an appealing alternative or adjunct to the more competitive summer slots [3].

It is unclear whether there are any grade implications of a later sub‐internship; limited data exists regarding seasonal variation in sub‐internship course outcomes. Prior studies suggest that students generally perform better on their home EM sub‐internship than their away rotation; however, evidence specific to away rotation timing and its impact on course outcomes is lacking [4, 5]. Retrospective studies and residency program director surveys have shown that EM sub‐internship grades are highly weighted in an applicant's ultimate ranking, underscoring the potential implications of any seasonal differences [6, 7, 8, 9].

By examining the course outcomes of medical students in their emergency medicine sub‐internship at one institution between 2021 and 2024, the objective of this study was to determine whether the time of year impacted a student's course grade.

## 2 Methods

### 2.1 Study Design and Setting

This was a retrospective observational study conducted in an urban mountain west environment. Rotation sites included a tertiary academic center and a tertiary community center. The rotation was 4 weeks long and consisted of an equal number of shifts at both the academic site and the community site. At the community site, students were evaluated by community emergency medicine attendings, while at the academic site students could be evaluated by academic emergency medicine attendings, fellows and residents.

### 2.2 Selection of Participants

All participants included in this study were medical students in an emergency medicine sub‐internship who had declared emergency medicine as their intended specialty.

### 2.3 Measurements and Data Collection

Data were collected via on‐shift evaluations that medical students received after their shift. The evaluation forms were provided via paper cards that a student handed physically to their evaluator (see Figure S1) or a QR‐code linked electronic evaluation form.

In these evaluation forms, students were graded in various subcategories of clinical competency, receiving scores ranging from 0 (inadequate) to 4 (outstanding). Notably, the evaluation forms did slightly change when the format was transitioned from paper to electronic. The evaluation subcategories switched from 11 categories (interviewing and physical examination skills, documentation, teaching point/patient presentation, ability to develop differential plan, procedures, resource utilization, clinical knowledge, professional attitude, response to instruction, patient interactions, working relationships) to 7 categories (patient interviewing, physical examination, clinical reasoning, clinical testing, documentation, presentation, inter‐professional teamwork), although the 0–4 grading schema remained the same. The average evaluation score of each card was averaged, and then contributed to the student's overall course grade.

A student's overall course grade was composed of 3 components; the average evaluation score (the evaluation scores received on shift), the SAEM exam score (online standardized exam each student took toward the end of their rotation), and an observed clinical encounter (in person graded clinical encounter with a clinical educator supervising). The overall course grade breakdown by component comprised 85% evaluation grade, 10% SAEM exam, and 5% observed clinical encounter. The course grade was also standardized to a scale of 0–4, with ≥ 3.5 receiving a grade of Honors, 3.0–3.4 being High Pass, 2.0–2.9 being Pass, and ≤ 1.9 receiving a grade of Fail.

### 2.4 Outcomes

The primary outcomes in this study were sub‐internship course outcomes, which included the average evaluation score (grade on a scale of 0–4), SAEM score (graded 0–100) and final course score (scale of 0–4), with the variable of interest being the rotation timing. The rotation season was categorized into two groups: May–July and August–October.

### 2.5 Analysis

Descriptive statistics were used to summarize the baseline and outcome variables. Continuous variables were reported as means with standard deviation or median with interquartile ranges, depending on their distribution, while categorical variables were summarized as frequencies and relative frequencies. When examining associations between rotation timing and outcome variables, independent *t*‐tests and Mann–Whitney *U* tests were used for normally distributed and non‐normally distributed variables, respectively. Multivariate linear regression analysis was then utilized to further investigate those associations and to adjust for any potential confounders. All the statistical tests were conducted using a significance level of 0.05. Data analyses were performed with SPSS software, version 29.

## 3 Results

A total of 84 students were included in this study, with 25 (29.8%) rotating between May and July, and 59 (70.2%) rotating between August and October. When comparing the course outcomes between these two groups, there was no statistically significant difference in average evaluation scores, SAEM scores, or final course grades (Table 1).

**TABLE 1:** Comparison of baseline characteristics and outcome variables by sub‐internship season: May–July and August–October.

Variable	Total (*N* = 84)	May–July 25 (29.8%)	August–October 59 (70.2%)	*p*
Gender				
Male	42 (50.0%)	15 (60.0%)	27 (45.8%)	0.17
Female	42 (50.0%)	10 (40.0%)	32 (55.2%)	
Season				
MD	65 (77.4%)	23 (92.0%)	42 (71.2%)	0.03
DO	19 (22.6%)	2 (8.0%)	17 (28.8%)	
Number of evaluations				
Median (IQR)	17 (13–21)	17.0 (13–20)	18.0 (13–21)	0.36
Overall evaluation score				
Mean ± SD	3.50 ± 0.27	3.45 ± 0.28	3.53 ± 0.27	0.11
SAEM score				
Mean ± SD	82.87 ± 6.03	83.19 ± 7.3	78.01 ± 5.44	0.32
Final score				
Mean ± SD	3.50 ± 0.25	3.49 ± 0.27	3.52 ± 0.24	0.21

## 4 Discussion

This study revealed that there was no significant difference in course outcomes when comparing early summer (May–July) to early fall (August–October) emergency medicine sub‐internships. These results suggest that student performance remains relatively stable across the peak months of the EM application season. For students navigating a competitive and often logistically constrained scheduling and application landscape, these findings provide reassurance that the timing of a sub‐internship does not markedly affect course outcomes. Stability in performance across the application season may reduce the stress and scheduling concerns for fourth‐year medical students as well as the logistical challenges for program leadership when planning sub‐internship rotations.

These findings add to the limited literature examining the timing of sub‐internship rotations in emergency medicine. Prior work has highlighted the importance of completing both home and away sub‐internships, as well as the potential advantages of early rotations in allowing students to obtain letters of recommendation before the residency applications are due [10, 11]. The implicit assumption is that ‘earlier is better’, with some studies specifically advising completion of one to two emergency medicine clerkships within the first few months of the fourth year [12]. Other advising resources are more vague, recommending that students complete their emergency medicine clerkships within the summer and fall of the fourth year without specifying precise months. Collectively, this literature reflects a tension between the assumed advantages of early rotations and the practical constraints that may limit student access to these opportunities.

Importantly, our results indicate that late summer and early fall rotations do not appear to disadvantage students in terms of course outcomes. For students, this finding may reduce anxiety about the implications of scheduling constraints, such as conflicts with board scheduling, required clerkships, travel, or other personal obligations. From an educational perspective, these findings may support residency and clerkship leadership in advising students on the timing of sub‐internship rotations; though notably these findings are based on data from a single institution.

Future studies including larger, multi‐institutional cohorts are needed to determine whether these findings can be generalized across different settings, specialties and geographic regions. Additionally, future work could explore other potential impacts of rotation timing, such as effects on standardized letters of evaluation, residency interview offers, or match outcomes, which are beyond the scope of this study.

## 5 Limitations

This study was conducted at a single academic center, which may limit the generalizability of these findings to other specialties or training environments. Additionally, the relatively small sample size limited our ability to perform more detailed sub‐group and seasonal analyses. Future studies with larger sample sizes may be able to further differentiate subtle differences in course outcomes on the basis of seasonal variations. Further research is needed to include other institutions across different geographic regions and training environments in order to enhance external validity and strengthen the opportunities for more granular data analysis. Additionally, future research is needed into other variables that may impact student evaluation scores such as the impact of academic vs. community faculty evaluations, rotation timing in terms of number of rotations completed by each student, and evaluation scores for home vs. away rotations.

## 6 Conclusions

There were no meaningful differences in early summer and fall rotation outcomes among students completing emergency medicine sub‐internships during peak application season. These findings offer reassurance to both students and educators that performance remains stable across the peak application season and could reduce concern that rotation timing alone influences course outcomes. As the single‐site nature of this research may limit current generalizability, multi‐institutional research is needed to validate these results and explore further relationships between rotation timing and other metrics such as standardized letters of evaluation, interview offers and match results.

## Author Contributions

**Sofia Tuttle:** conceptualization, data curation, project administration, writing – original draft, writing – review and editing. **Christine Raps:** conceptualization, methodology, writing – review and editing, investigation. **Emad Awad:** methodology, data curation, formal analysis, writing – review and editing. **Patrick G. Hughes:** conceptualization, methodology, supervision, visualization, project administration, writing – original draft, writing – review and editing. **Rowan Kelner:** conceptualization, data curation, formal analysis, writing – review and editing. **Allison Beaulieu:** conceptualization, methodology, investigation, supervision, project administration, writing – review and editing. **Robert Stephen:** conceptualization, methodology, writing – review and editing. **Brian Merritt:** methodology, conceptualization, writing – review and editing. **Julia Ruggieri:** conceptualization, methodology, writing – review and editing.

## Funding

The authors have nothing to report.

## Conflicts of Interest

The authors declare no conflicts of interest.

## Supporting information


**Figure S1:** Evaluation form.

## Data Availability

The data that support the findings of this study are available on request from the corresponding author. The data are not publicly available due to privacy or ethical restrictions.
