# Supplementary material for: Impact of Rotation Timing on Emergency Medicine Sub‐Internship Course Outcomes
Source: AEM Educ Train. 2026 Jul 23;10(4):e70227. doi: 10.1002/aet2.70227 (PMC13396426; doi:10.1002/aet2.70227)

**Figure 1S – Evaluation Form**

Pictured below is the medical student evaluation form (front and back) used to collect feedback from residents and attendings during a medical student’s shift.


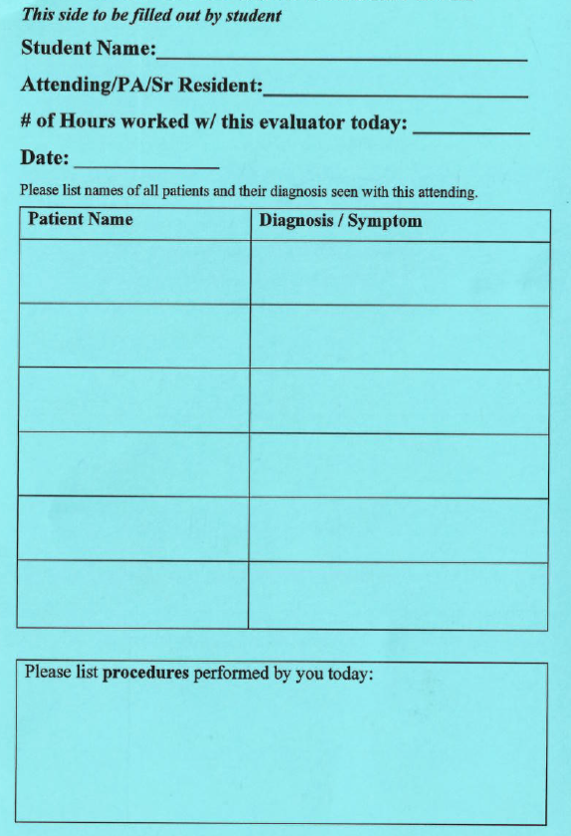

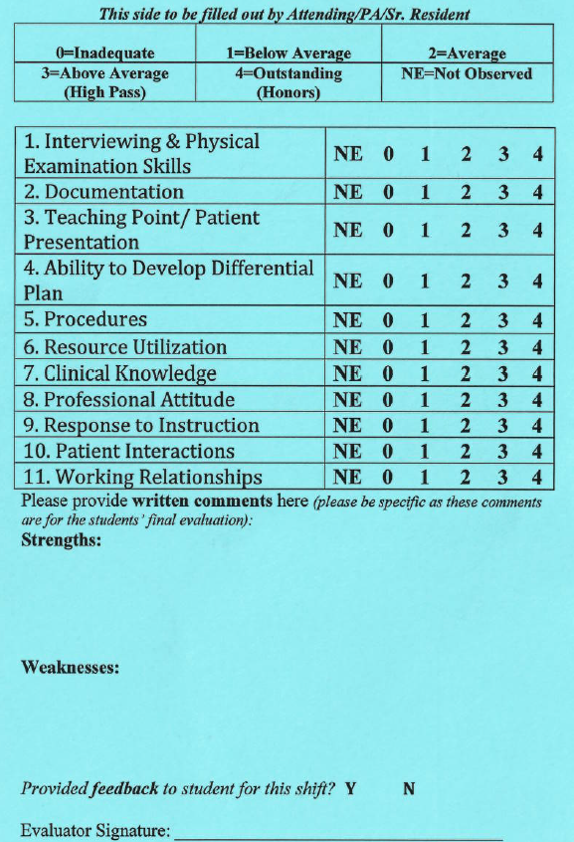

Supplement: Supplementary file 1 — Figure S1: Evaluation form. [file AET2-10-e70227-s001.docx]
